# Advances in adhesion-related pathogenesis in *Mycoplasma pneumoniae* infection

**DOI:** 10.3389/fmicb.2025.1613760

**Published:** 2025-07-23

**Authors:** Bingyue Sun, Yaozheng Ling, Junhui Li, Li Ma, Zige Jie, Hongbing Luo, Yang Li, Guo Yin, Mingwei Wang, Fanzheng Meng, Man Gao

**Affiliations:** ^1^Department of Pediatric Respiration, Children’s Medical Center, The First Hospital of Jilin University, Changchun, China; ^2^Department of Developmental and Behavioral Pediatrics, The First Hospital of Jilin University, Changchun, China; ^3^The First Hospital of Jilin University, Changchun, China; ^4^NHC Key Laboratory of Radiobiology, School of Public Health, Jilin University, Changchun, China; ^5^Center for Pathogen Biology and Infectious Diseases, The First Hospital of Jilin University, Changchun, China

**Keywords:** *M. pneumonia*, terminal organelle, adhesion, treatment, vaccines

## Abstract

*Mycoplasma pneumoniae* is a leading cause of community-acquired pneumonia (CAP) and upper respiratory tract infections, particularly in children and immunocompromised individuals. The growing global prevalence of macrolide-resistant *M. pneumoniae* (MRMP) further emphasizes the urgent need to elucidate its pathogenic mechanisms. Among these, adhesion plays a central role, serving as a prerequisite for colonization and disease progression, and thus warrants detailed investigation. The terminal organelle of *M. pneumoniae* mediates both adhesion and gliding motility, facilitating colonization, tissue invasion, and potential systemic spread. In the lung, adhesion triggers cytotoxic effects through the release of hydrogen peroxide (H_2_O_2_) and CARDS toxin (CARDS TX), promotes excessive inflammatory responses, and enables immune evasion via antigenic variation. Extrapulmonary manifestations may also arise either from direct bacterial dissemination or autoimmune responses induced by molecular mimicry between bacterial and host antigens. In addition, recent advances suggest that therapies and vaccines directed at the adhesion mechanism of *M. pneumoniae* may offer promising strategies for combating MRMP infections. Although progress has been made, the adhesion-related pathogenesis of *M. pneumoniae*, as well as the prospects for therapies and vaccines targeting this mechanism, remains incompletely defined. This review synthesizes current insights into adhesion-mediated mechanisms and highlights emerging therapeutic strategies targeting adhesion, aiming to support more effective treatment and prevention of *M. pneumoniae* infection.

## 1 Introduction

*Mycoplasma pneumoniae* (*M. pneumoniae*), a minimalist bacterial pathogen with a streamlined genetic architecture, possesses one of the smallest genomes among free-living organisms, spanning about 816 kilobase pairs (kbp). This genomic reduction reflects its evolutionary adaptation to obligate parasitism in the human respiratory tract and makes it a valuable model for studying host–pathogen interactions in genome-limited microbes (Kumar, 2018). Despite lacking a cell wall, *M. pneumoniae* has evolved a structurally optimized triple-layered membrane enriched with sterols and transmembrane proteins, which confers intrinsic resistance to β-lactam antibiotics that target peptidoglycan synthesis (Meseguer et al., 2003). Its genomic core houses essential macromolecular complexes, including a covalently closed circular DNA genome, RNA polymerase (RNAP), ribosomes, and mRNAs, which collectively drive its streamlined transcriptional-translational machinery. Critically, the transcription elongation factor N-utilizing substance A (NusA) mediates dynamic coupling between RNAP and ribosomes through direct physical interactions, synchronizing transcription with translation to ensure rapid gene expression (Meseguer et al., 2003; Liang et al., 2012; O’Reilly et al., 2020).

*M. pneumoniae*, which is globally distributed, is a common cause of community-acquired pneumonia (CAP), which accounts for about 10–30% of all CAP cases and upper respiratory tract (URT) infections, particularly in children and immunocompromised adolescents (Yoon et al., 2017). Although *M. pneumoniae* pneumonia (MPP) is considered a self-limiting disease, it causes various pulmonary symptoms, such as fever, dry cough, dyspnea, and wheezing, along with several complications, such as atelectasis and pleural effusion (Hawkins et al., 2011; Zhang et al., 2020). Additionally, refractory *M. pneumoniae* pneumonia (RMPP) has been increasingly reported and may be accompanied by severe necrotizing pneumonia, bronchitis obliterans, thrombosis, etc. RMPP is more common in children than in adults, with a rising incidence rate (Liu et al., 2020; Zhai et al., 2020). These infections not only impose a considerable medical burden on humans but can also induce severe sequelae, such as bronchiolitis obliterans, atelectasis, and bronchiectasis (Huang et al., 2018). *M. pneumoniae* infection also causes a wide range of extrapulmonary diseases involving multiple systems, including the cutaneous, musculoskeletal, neurological, hematological, digestive, and renal systems (Poddighe, 2018; Hu et al., 2022). Meanwhile, the widespread prevalence of macrolide resistant *M. pneumoniae* (MRMP) poses a great challenge to resist *M. pneumoniae* (Jiang F. C. et al., 2021). To better understand and treat MPP/RMPP-related diseases and other extrapulmonary diseases caused by *M. pneumoniae*, we aimed to further elucidate the pathogenesis of *M. pneumoniae*.

According to previous research, the possible pathogenesis of *M. pneumoniae* includes adhesion damage, destruction of membrane fusion, nutrient depletion, toxic injuries, inflammatory injuries, and other immune-related injuries (He et al., 2016; Yiwen et al., 2021). Among these, pathogen adhesion to host cells is a critical virulence factor, facilitating microbial colonization by evading mucociliary clearance and immune surveillance, and establishing the basis for subsequent invasion (Vaca et al., 2020). Specifically, the adherence of *M. pneumoniae* to the respiratory epithelium is a key step in initiating infection, allowing the release of cytotoxins, tissue damage, and immune evasion (Kashyap and Sarkar, 2010).

Despite its critical role in infection, the precise pathogenesis involving *M. pneumoniae* adhesion remains elusive. Therefore, this review systematically synthesizes current findings on the role of adhesion mechanisms in *M. pneumoniae*-host interactions and outlines future research directions to support the development of improved prevention and treatment strategies.

## 2 Adhesion and gliding mechanisms of *M. pneumoniae*

### 2.1 The adhesion mechanisms of *M. pneumoniae*

#### 2.1.1 Structure of the terminal organelle

**FIGURE 1:**
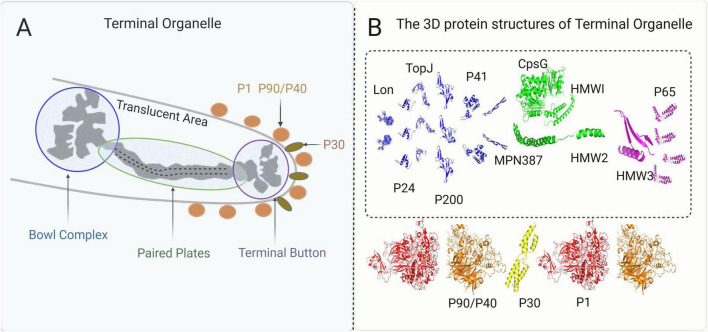
**(A)** A pattern map of the terminal organelle’s structures. **(B)** The structures of the proteins in the terminal organelle and their relative positions. The 3D structures of the proteins are visualized by PyMOL software based on their amino acid sequences from NCBI, while their relative positions are described based on the core image and recent mapping results from Hamaguchi (2016).

The terminal organelle of *M. pneumoniae*, also referred to as the attachment organelle or tip structure (Miyata and Hamaguchi, 2016), orchestrates both cytoadherence to host epithelia and gliding motility, which are essential for tissue dissemination (Krause et al., 2018; Widjaja et al., 2020). This specialized polar membrane protrusion has a bipartite architecture: surface-exposed nap-like proteins facilitate host-pathogen interactions, and an intricately organized internal structure enables the generation of mechanical force (Nakane et al., 2015). The adhesion machinery of terminal organelles comprises four evolutionarily conserved surface proteins that mediate gliding and adhesion mechanisms: P1 (MPN141), P90/P40 (encoded by MPN142 as proteolytic cleavage products), and P30 (MPN453). Spatial mapping has demonstrated that the P1 adhesin complex, comprising the P1 and P90/P40 subunits, is strategically localized at the apical tip of the organelle, forming a rigid membrane anchor. Whereas P30 dynamically associates with the complex periphery to regulate force transduction during gliding motility (Nakane et al., 2015; Miyata and Hamaguchi, 2016; Zuo et al., 2024). Internally, the terminal organelle exhibits a sophisticated internal architecture comprising an electron-dense core structure that maintains structural integrity through three specialized components: the terminal button, paired plates, and bowl complex. This core is enveloped by a translucent matrix region. This highly organized core scaffold provides mechanical stability and serves as an assembly platform for the adhesion machinery. The terminal button, situated within the inner peripheral membrane region, is primarily composed of three core proteins: P65 (MPN309), HMW2 (MPN310), and HMW3 (MPN452). Adjacent to this structure, the paired plates system has a stratified organization consisting of four distinct components: HMW1 (MPN447), HMW2 (MPN310), CpsG (MPN066), and HMW3 (MPN452). The paired plates exhibit distinct protein compositions across their morphological subtypes: the thin plates are primarily composed of HMW1 and CpsG, whereas the thick plates are enriched in HMW2. The plate complex occupies a posterior position within the organelle architecture, where the thick and thin plates form an integrated unit through direct adhesion. This composite structure establishes stable connections with bowl-shaped structural elements through precise molecular interactions. Structural analysis of the coiled HMW2 protein revealed critical functional domains: its N-terminal region mediates attachment to the terminal button complex, whereas the C-terminal domain facilitates integration with the bowl structure. This bipolar molecular configuration suggests that HMW2 serves as a key architectural element that bridges distinct organellar components (Kawamoto et al., 2016). The bowl complex itself is composed of seven core components: TopJ (MPN119), P24 (MPN312), Lon protease (MPN332), P200 (MPN567), MPN387, P41 (MPN311), and HMW2 (MPN310) (Nakane et al., 2015; Miyata and Hamaguchi, 2016). MPN387 is specifically localized at the interface between the plates and bowl, suggesting a potential coordinating function. The translucent area occupied by a rigid, electronically transparent substance transmits the force generated by the bowl complex to paired plates (Balish et al., 2003; Kawamoto et al., 2016; Figure 1). As discussed above, the terminal organelle is a highly organized and functionally coordinated structure, and the specific roles of its components are further detailed below.

#### 2.1.2 Adhesion mechanisms of the terminal organelle

Sialylated oligosaccharides (SOS) are the terminal glycan modifications that cap the outer branches of glycoconjugates on cell surface-associated glycoproteins and glycolipids. They also serve as essential structural components of mucosal glycoproteins within mucus layers (Miyata, 2008; Kasai et al., 2013; Balish, 2014). Glycoproteins, a class of macromolecules ubiquitously present in biological systems, are strategically localized on the plasma membranes, within the extracellular matrix, and throughout bodily fluids. They exhibit functional diversity across processes such as molecular recognition, immune modulation, and intercellular communication. Among these functions, cell adhesion is primarily mediated by two major families of glycoproteins: integrins and cadherins (Chastney et al., 2025; Jiang et al., 2025). In microbial infection, SOS, which serve as key receptor molecules on the surface of host cells and within the mucus layer, are specifically recognized by a variety of pathogenic microorganisms and exploited to facilitate adhesion and invasion (Table 1; Spahich and St Geme, 2011; Hentrich et al., 2016; Andreae et al., 2018; Marc et al., 2018; Hong et al., 2022; Pronker et al., 2023; Aberg et al., 2024; Ni et al., 2024). Similarly, *M. pneumoniae* utilizes the terminal organelle to bind to SOS in a “lock-and-key” pattern to initiate its adhesion. Both α-2,6- and α-2,3-sialyllactose support the adherence of *M. pneumoniae*, of which α-2,3-sialyllactose has a relatively high affinity (Williams et al., 2020; Vizarraga et al., 2021). The interaction between *M. pneumoniae* and SOS on glycoproteins distributed across multiple organs and tissues may represent a fundamental pathogenic mechanism contributing to the multi-system involvement in *M. pneumoniae* infections.

**TABLE 1:** Microbial pathogens employ adhesion strategies to establish host colonization.

Species	Name	Mechanism
Bacteria	*Hemophilus influenzae*	Binding of surface adhesin proteins (Hia, Hsf) to the SOS in host respiratory epithelial cells to mediate respiratory colonization.
	*Streptococcus pneumoniae*	Secretory neuraminidase (NanA) cleaves sialic acid residues, exposing underlying receptors to promote colonization.
	*Neisseria meningitidis*	Using outer membrane proteins Opa and OpC to bind to host receptors, such as sialated CD46, to promote blood-brain barrier crossing.
	*Helicobacter pylori*	SabA adhesin specifically recognizes sLex/a in the gastric mucosal layer to mediate gastric epithelial adhesion and chronic infection.
Virus	*Influenza virus*	Hemagglutinin (HA) protein recognizes the sialic acid receptor on the host cell surface to initiate infection.
	*SARS-CoV-2*	Indirectly promoting membrane fusion through histone proteins released by neutrophils, linking the spike protein and sialic acid.
Fungi	*Candida albicans*	Surface lectin-like sequence (ALS) family proteins bind to host sialoglycans to promote biofilm formation and tissue invasion.

In *M. pneumoniae*, multiple adhesin proteins localized within the terminal organelle contribute to its attachment to host cells. Among them, P1 functions as the primary adhesin and plays a central role in mediating the bacteria-host interaction. Strains lacking P1 entirely lose their adhesion capability and consequently become non-pathogenic. Through the action of the P1 adhesin, *M. pneumoniae* attaches to host epithelial cells and localizes within intercellular spaces, thereby evading ciliary clearance and macrophage phagocytosis. This allows the pathogen to release virulence factors and inflict damage on host tissues (He et al., 2016; Widjaja et al., 2020). Although P1 adhesin is an essential component for both adhesion and gliding, it can mediate binding between *M. pneumoniae* and host receptors only when correctly localized on the terminal organelle (Widjaja et al., 2020). One accessory protein, the DnaJ-like chaperone TopJ, which contains a J-domain, an acidic- and proline-rich region (APR), and a C-terminal domain, efficiently facilitates the delivery of P1 to the surface of the terminal organelle (Cloward and Krause, 2010, 2011). In addition, the P90/P40 adhesins of *M. pneumoniae* also exhibit specific binding affinity to the terminal sialylated glycans of host oligosaccharides. P90/P40 proteins associate with P1 adhesin to form an adhesin complex that further enhances the adhesive properties of *M. pneumoniae* (Kenri et al., 2008; Yamazaki and Kenri, 2016; Kenri et al., 2019; Vizarraga et al., 2020; Marseglia et al., 2024). P30, another membrane protein, has been shown to localize at the distal end of the terminal organelle. Research suggested that P30, with a homologous sequence of P1 in a certain domain, implies a potential beneficial association for adhesion (Miyata and Hamaguchi, 2016). Notably, P30 and P65 exhibit a close functional and spatial relationship, wherein P65 interacts with the internal domain of P30 to mediate precise contact between the terminal button and the frontal region of the structural layer (Miyata and Hamaguchi, 2016). Furthermore, the P41 protein governs the polar localization of the terminal organelle and directs the positioning of P24 within the adhesion complex. Together, P41 and P24 ensure proper spatial organization of the adhesin machinery, thereby facilitating effective host adhesion (Hasselbring and Krause, 2007; Krause et al., 2018).

Additionally, the HMW1, HMW2, and HMW3 proteins can also contribute to the adhesion of *M. pneumoniae* to the airway epithelium (Page and Krause, 2013). The loss of HMW1 results in a deletion at the 3’ end of the p30 gene and disrupts the functional association among HMW2, HMW3, and P65. This disruption consequently impairs the clustering of P1 at the cell’s polar end, thereby weakening the adhesive capacity. Furthermore, dysfunction of HMW3 suppresses P65 expression and causes its diffuse localization, preventing proper positioning of the P1 adhesin at the terminal organelle and further reducing adhesion efficiency (Hahn et al., 1998; Willby and Krause, 2002).

#### 2.1.3 Adhesion mechanisms of adherence factors

While the terminal organelle is essential for primary adherence, *M. pneumoniae* also expresses adhesion factors that function independently of this structure. Notably, several surface-exposed glycolytic enzymes have recently been identified as non-classical adhesins involved in host-pathogen interactions (Grundel et al., 2015). These enzymes, including lactate dehydrogenase (LDH), phosphoglycerate mutase (PGM), pyruvate kinase (PYK), glyceraldehyde-3-phosphate dehydrogenase (GapA), transketolase (TKT), and pyruvate dehydrogenase subunits A to C (PdhA-C), have been shown to interact with components of the extracellular matrix (ECM), thereby contributing to both adhesion and invasion processes of *M. pneumoniae* (Thomas et al., 2013; Gründel et al., 2015; Grundel et al., 2016; Grimmer and Dumke, 2019). In addition, elongation factor Tu (MPN665, EF-Tu), a well-known moonlighting protein with multiple non-overlapping functions, has been identified on the surface of *M. pneumoniae* in several studies (Dallo et al., 2002; Balasubramanian et al., 2008). Besides its canonical role in cytoplasmic biosynthesis and metabolism, EF-Tu has also been shown to bind fibronectin (Fn), thereby mediating the interaction between *M. pneumoniae* and the ECM (Dallo et al., 2002; Balasubramanian et al., 2008; Grimmer and Dumke, 2019). The ECM consists of two major components: the interstitial connective tissue matrix, which provides structural support, and the basement membrane, a specialized layer that regulates cellular organization and differentiation (Karamanos et al., 2021). Interactions between host cells and the ECM are primarily mediated by laminin (Ln) within the basement membrane, as well as by interstitial matrix proteins such as Fn, vitronectin (Vn), and fibrinogen (Grundel et al., 2016). Integrins, a ubiquitous family of transmembrane receptor proteins expressed on human cells, recognize Arg-Gly-Asp (RGD) motifs within these ECM components and facilitate cellular anchorage through bidirectional mechanotransduction (Sutherland et al., 2023). When surface-associated glycolytic enzymes of *M. pneumoniae* bind to these ECM proteins, they function as bacterial ligands that interact with host cell receptors, thereby enhancing the adhesion of *M. pneumoniae* to respiratory epithelial cells (Bao et al., 2023; Pang et al., 2023).

#### 2.1.4 Focal adhesion structure

**FIGURE 2:**
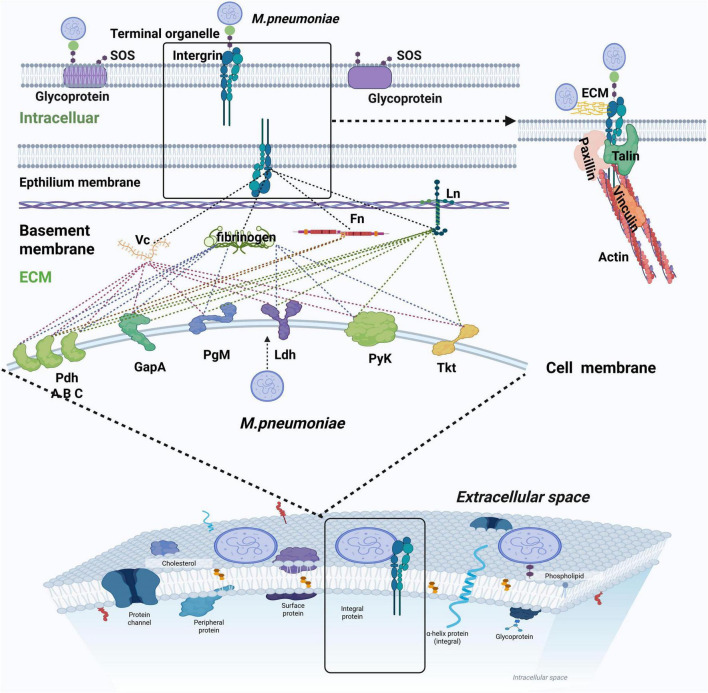
*M. pneumoniae* employs specialized adherence organelles and glycolytic enzymes to establish adhesion to respiratory epithelial cells. Notably, distinct glycolytic enzymes mediate host cell attachment by interacting with different ECM proteins, as indicated by dashed lines in the schematic representation (Grundel et al., 2016). When adhering to integrins, the pathogen forms an FA-like structure. This structure achieves mechanical stability through adaptor proteins, including talin, vinculin, and paxillin, which anchor the adhesion complex to intracellular actin filaments, creating a robust host-pathogen interface.

Whether *M. pneumoniae* utilizes the terminal organelle or surface-associated glycolytic enzymes for adhesion, integrins on the host cell membrane play a central role in mediating this interaction. When *M. pneumoniae* adhesins engage with integrins, they initiate the assembly of “focal adhesion” (FA) complexes. FA anchors to intracellular actin filaments through adaptor proteins such as talin, vinculin, and paxillin, establishing a stable adhesion interface (Kanchanawong and Calderwood, 2023; Matrullo et al., 2025). This integrin-mediated FA structure has been demonstrated to be critical for static adhesion during host-pathogen interactions (Figure 2; Jones et al., 2019; Kamranvar et al., 2022).

### 2.2 Gliding motility based on the adhesion mechanism

The absence of a cell wall in *M. pneumoniae* facilitates its evolution of a unique translocation mechanism known as gliding motility (Miyata and Hamaguchi, 2016). Both adhesion and gliding can occur on the mucus layer and ciliated epithelial cells. Confocal microscopy studies using normal human bronchial epithelial (NHBE) cells have demonstrated that once *M. pneumoniae* traverses the mucus layer, it is capable of gliding along the cilia, migrating toward their base, and ultimately reaching the host cell surface (Prince et al., 2014).

**FIGURE 3:**
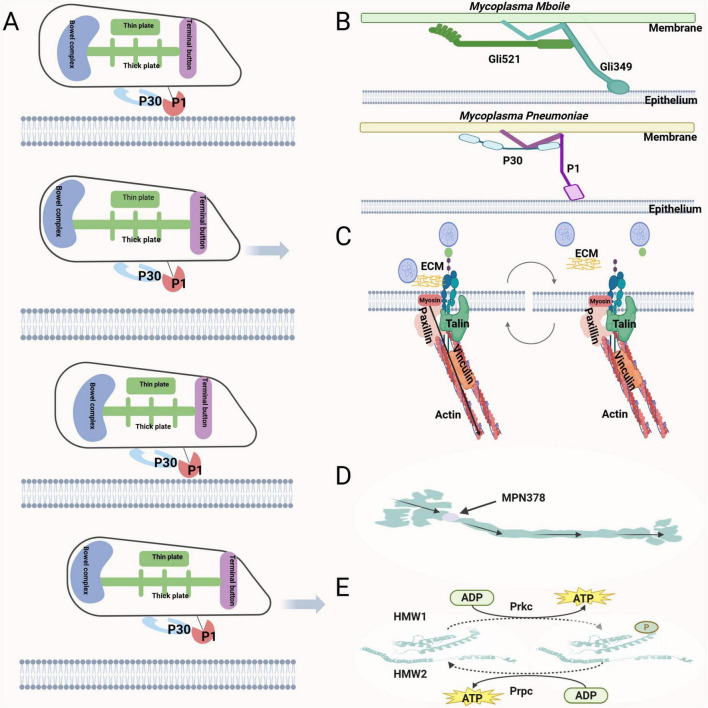
**(A)** The gliding mechanism of *M. pneumoniae*. P1 adhesin binds to the host cell surface through a catch-pull-release cycle with SOS. During the terminal organelle’s extension, P1 retracts from SOS, and during contraction, P1 binds to SOS tightly, while the iterative extension and retraction of the terminal organelle generate gliding movement. **(B)** The P1 and P30 proteins of *M. pneumoniae* may exhibit functional homology to the Gli349 and Gli521 adhesins in *M. mobile*, suggesting conserved molecular mechanisms underlying mycoplasma motility. **(C)** From the host’s perspective, when the integrin binds to the substrate, myosin generates force, actin contracts, and the substrate detaches, thereby promoting the sliding of *M. pneumoniae* on the cell membrane. **(D)** The internal force direction of the core structure. The terminal organelle forces originate in the bowl complex, travel through MPN387 to the paired plates, and then lead to extension and retraction of the terminal organelle. **(E)** PrkC promotes the phosphorylation of HMW1 and HMW2 proteins to enhance *M. pneumoniae* gliding motility, whereas PrpC functions opposite manner.

The terminal organelle of *M. pneumoniae* interacts with SOS on the host cell surface to form a gliding fulcrum (Kawamoto et al., 2016). The pathogenic role of P1 in *M. pneumoniae* is thought to stem primarily from its function in mediating static adhesion during gliding. Specifically, the P1 adhesin binds to SOS on the host cell surface via a catch-pull-release mechanism, which is coordinated with the repeated extension and contraction of the terminal organelle to generate directional gliding. Additionally, the paired plates within the terminal organelle are essential for motility. They facilitate elongation and contraction of the organelle through structural remodeling in the distal region of the thick plate. During extension of the terminal organelle, the P1 adhesin detaches from SOS, whereas during contraction, it rebinds tightly (Kawamoto et al., 2016). Thus, gliding is achieved through iterative cycles of binding and release (Figure 3A). After the use of monoclonal antibodies against P1 adhesin, the gliding speed decreased over time, and gliding cells were eventually removed from the glass surface (Seto et al., 2005). Emerging evidence suggests that the P30 protein in *M. pneumoniae* may function as a rotational-to-linear mechanotransducer, functionally analogous to the Gli521 “crank” protein in *Mycoplasma mobile* (*M. mobile*). This proposed mechanism involves the conversion of ATP hydrolysis-driven rotational torque into directional movement through coordinated interactions with the P1 adhesin, which is proposed to function as a homolog of the Gli349 “leg protein” in *M. mobile’s* gliding apparatus (Figure 3B; Toyonaga et al., 2021; Zuo et al., 2024). From the host’s perspective, during *M. pneumoniae* adhesion to integrin receptors, the host cell initiates a mechanotransduction cascade: myosin generates tension, triggering the retrograde flow of actin filaments and the disassembly of focal adhesions. This coordinated cytoskeletal remodeling creates a propulsive substrate deformation wave, enabling directional gliding of the pathogen along the membrane plane through force-coupled membrane lipid redistribution. Force conduction throughout the entire process is directly completed by the talin protein and indirectly by the vinculin and paxillin proteins (Figure 3C; Matrullo et al., 2025).

The internal core of the terminal organelle is essential for maintaining gliding motility in *M. pneumoniae*. Earlier studies have shown that P200, MPN387, and TopJ proteins around the organelle base are more related to gliding than binding (Hasselbring et al., 2005; Pich et al., 2006; Jordan et al., 2007; Nakane et al., 2015). Thus, it is currently believed that the mechanical forces driving the terminal organelle originate from the bowl complex, travel through MPN387 to the paired plates, and subsequently lead to extension and retraction of the terminal organelle, finally causing P1 to attach to the internal core to undergo a corresponding catch-pull-release cycle (Figure 3D; Nakane et al., 2015; Kawakita et al., 2016; Kawamoto et al., 2016). In terms of the research above, the effective transmission of internal force plays a pivotal role in *M. pneumoniae* adhesion and gliding motility. Phosphoprotein staining has confirmed a correlation between the phosphorylation of cytoskeletal proteins HMW1 and HMW2 and gliding activity. Since HMW1 and HMW2 are phosphorylated in an ATP-dependent manner by Ser/Thr kinases, the gliding characteristic can be partially controlled by reversing Ser/Thr phosphorylation, thereby affecting gliding frequency. The Ser/Thr protein kinase PrkC (MPN248) and its cognate phosphatase PrpC (MPN247) exert opposing effects on the gliding frequency of *M. pneumoniae*. PrkC acts as a phosphokinase, promoting the phosphorylation of HMW1 and HMW2 proteins to enhance motility; however, PrpC acts antagonistically by dephosphorylating these proteins, thereby reducing gliding activity (Figure 3E; Page and Krause, 2013).

### 2.3 Comparative analysis of adhesion mechanisms between *M. pneumoniae* and *M. genitalium*

To better elucidate the adhesion-related pathogenic mechanisms of *M. pneumoniae*, the adhesion mechanisms of *Mycoplasma genitalium* (*M. genitalium*), another phylogenetically related member of the Mollicutes class that possesses highly conserved adhesion strategies, were referred to and investigated. Similar to *M. pneumoniae*, *M. genitalium* utilizes a terminal organelle for adhesion, with core adhesins being P140 (MgPa) and P110 (MgPc) proteins (Deng et al., 2018). MgPa, the major adhesin, has been shown to bind to host molecules such as cyclosporin A (CypA) and histone H2B (Liao et al., 2021; Li et al., 2020). P32, a protein homologous to the P30 adhesin of *M. pneumoniae*, is essential for maintaining the stability of both MgPa and MgPc (Yueyue et al., 2022). Additionally, several accessory proteins, such as MG218, MG317, MG312, and P69, have been implicated in facilitating the adhesion process (Yueyue et al., 2022). *M. genitalium* also exhibits gliding motility, although its mechanism remains less well characterized than that of *M. pneumoniae*. Several adhesion-associated proteins, including MG218, MG317, and MG312, are involved in the gliding process, and gliding-specific proteins such as MG200 and MG386 are also indirectly linked to adhesion (Yueyue et al., 2022). Furthermore, CD14, a human receptor, recognizes lipid-associated membrane proteins (LAMPs) from *M. genitalium*, enhancing the release of TNF-α and intensifying the host inflammatory response (He et al., 2014).

Taken together, *M. genitalium* and *M. pneumoniae* share highly similar adhesion strategies, involving terminal organelles, coordinated binding-gliding dynamics, and downstream inflammatory cascades. Interestingly, while *M. genitalium* is typically associated with chronic urogenital infections, *M. pneumoniae* is more commonly linked to acute respiratory infections. Following adhesion, *M. genitalium* invades urogenital epithelial cells and can persist intracellularly for extended periods, promoting chronic infection. Antigenic variation of key adhesins such as MgPa and MgPc enhances immune evasion, further contributing to its ability to establish long-term colonization (McGowin and Totten, 2017; Yueyue et al., 2022). Investigating how *M. genitalium* utilizes adhesion to sustain persistent infection may provide critical insights into potential “stealth survival” strategies employed by *M. pneumoniae* within the respiratory tract.

### 2.4 Brief summary of adhesion and gliding mechanisms

*M. pneumoniae* adhesion is primarily mediated by the terminal organelle and associated adhesins, notably P1, through a “lock-and-key” interaction with SOS on the host cell surface. Adhesion-related proteins interact with host components to form FA complexes that further stabilize the attachment. Gliding motility, which is closely tied to adhesion, also utilizes the terminal organelle as a pivot point. P1 plays a critical role in this process by facilitating movement through a coordinated grab–pull–release mechanism, synchronized with the extension and retraction of the organelle. Together, adhesion and gliding on host surfaces represent essential steps in the pathogenic process of *M. pneumoniae*.

## 3 Adhesion-related mechanisms in intrapulmonary infection

### 3.1 Toxicity pathogenic mechanisms related to adhesion

Waites et al. (2017) demonstrated that *M. pneumoniae* adheres to epithelial cell surfaces to acquire essential nutrients while simultaneously releasing cytotoxic molecules that contribute to host cell damage. Upon adhesion, the pathogen extends microtubule-based structures into host cells, promoting metabolic exploitation by consuming oxygen, depleting glucose, absorbing cholesterol, and acquiring amino acids. These activities collectively lead to the accumulation of toxic metabolites and subsequent cellular injury (Schomburg and Vogel, 2012; Grosshennig et al., 2013; Kumar, 2018). Nevertheless, the precise molecular mechanisms underlying post-adhesion cytotoxicity and systemic tissue damage remain to be fully elucidated.

#### 3.1.1 Hydrogen peroxide

The adhesion of *M. pneumoniae* to host cells initiates a cascade of events critical for bacterial survival and persistence. A key pathogenic strategy involves the production of hydrogen peroxide (H_2_O_2_), which induces cytoskeletal reorganization by modulating effector proteins. This process depletes host nutrients and elevates cellular oxygen demand. Importantly, *M. pneumoniae* undergoes metabolic adaptation by utilizing host-derived glycerol as a carbon and energy source. Enhanced aerobic respiration leads to the accumulation of cytotoxic levels of H_2_O_2_ (Blotz and Stulke, 2017). Cytoskeletal reorganization of host epithelial cells appears pivotal in producing cytotoxic substances and facilitating bacterial dissemination. Notably, the adhesion of *M. pneumoniae* not only triggers cytoskeletal rearrangement but also facilitates its translocation across the bronchial mucosal barrier. This process is accompanied by sustained H_2_O_2_ release, leading to progressive pathological alterations in bronchial epithelial cells, including cellular edema, necrosis, intercellular adhesion, decelerated ciliary motility, and structural deformation (Blotz and Stulke, 2017). Although the precise mechanisms by which *M. pneumoniae* induces cytoskeletal remodeling remain incompletely defined, current evidence indicates that FA-mediated mechanotransduction plays a pivotal role. Specifically, pathogen adhesion activates focal adhesion kinase (FAK) and Src family kinases (Matrullo et al., 2025), which subsequently initiate Rho GTPase signaling cascades that drive cytoskeletal reorganization (Bement et al., 2024). Recent findings further suggest that force transmission from FAs to the cytoskeleton may represent the proximal mechanical signaling event initiating these structural changes. Notably, such mechanochemical coupling appears transient, potentially explaining the dynamic nature of cytoskeletal rearrangement during infection (Bement et al., 2024; Matrullo et al., 2025).

In this context, Glycerol-3-phosphate (G3P) serves as a critical carbon source at the adhesion site of *M. pneumoniae*. It is derived from free glycerol, which is transported into the cytoplasm via the glycerol facilitator (GlpF) and subsequently phosphorylated by glycerol kinase (GlpK). The lipoproteins MPN133 and MPN284 might also participate in the delivery of glycerol to GlpF. Additionally, glycerophosphodiesterase (GlpQ) converts glycerophosphocholine (GPC) into an alternative carbon source, which is imported via the recently identified transport protein GlpU (MPN421) (Halbedel et al., 2007; Grosshennig et al., 2013). The metabolism of G3P by glycerol-3-phosphate oxidase (GlpD) produces H_2_O_2_, which benefits bacterial survival but harms the host (Schmidl et al., 2011; Grosshennig et al., 2013).

**FIGURE 4:**
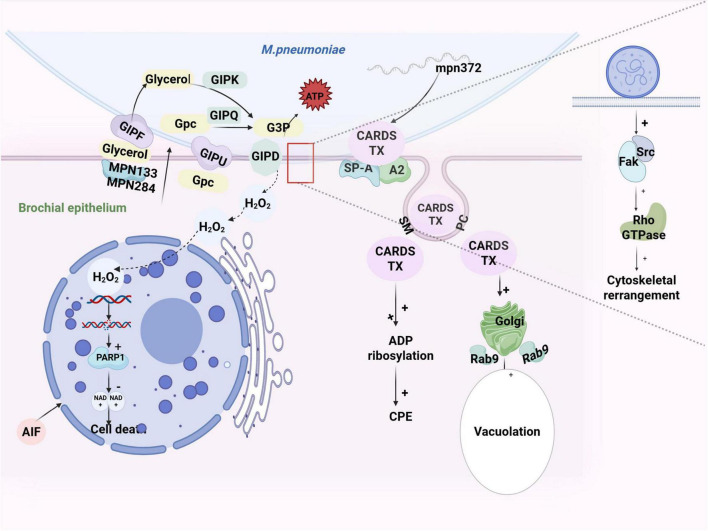
The adhesion-associated toxin mechanism of *M. pneumoniae*, including H_2_O_2_ and CARDS TX.

As a virulence factor, H_2_O_2_ plays a critical role in eliciting an oxidative stress response during *M. pneumoniae* infection. Due to the inadequacy of key antioxidant enzymes, such as superoxide dismutases and catalases, *M. pneumoniae* cannot effectively detoxify H_2_O_2_. As a major reactive oxygen species (ROS), pathologically accumulated H_2_O_2_ induces cellular oxidative stress, damages biomacromolecules, disrupts energy metabolism, and activates inflammatory responses (Park, 2013). For *M. pneumoniae*, recent studies have shown that H_2_O_2_ can accelerate the shedding of *M. pneumoniae*-infected cells. H_2_O_2_-induced oxidative DNA damage, including base oxidation and single- and double-strand breaks, triggers hyperactivation of poly (ADP-ribose) polymerase 1 (PARP1). This excessive DNA repair response depletes cellular nicotinamide adenine dinucleotide (NAD^+^) reserves, subsequently impairing mitochondrial ATP production. The ensuing bioenergetic collapse drives cells into a parthanatos death pathway, a PARP1-dependent programmed necrotic process characterized by irreversible metabolic failure (Yamamoto et al., 2019). ADP-ribosylation is a biochemical process in which the ADP-ribosyl group is transferred from NAD+ to specific amino acid residues on target proteins by ADP-ribosyl transferase (ADPRT). This reaction is catalyzed by several bacterial exotoxins, which disrupt macromolecular function and homeostasis (Krueger and Barbieri, 1995). Such disruptions facilitate the translocation of apoptosis-inducing factor (AIF) to the nucleus, ultimately resulting in apoptosis and cell death. Although the shedding of infected cells can reduce the infectivity of *M. pneumoniae* (Liesman et al., 2014), another study suggested that *M. pneumoniae* mitigates exogenous H_2_O_2_-induced shedding of infected epithelial cells by closely adhering to host cells and depleting cytosolic NAD^+^, which serves as the substrate for PARP1 activity (Figure 4; Yamamoto et al., 2019).

#### 3.1.2 CARDS TX

**FIGURE 5:**
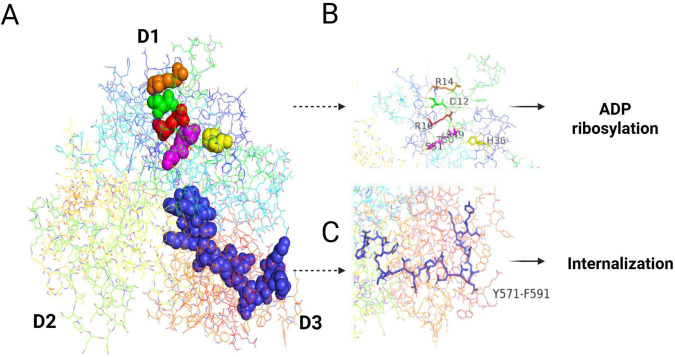
The CARDS TX structure mapped by PyMoL with the main pathogenic mechanism. **(A)** The 3D protein structure of CARDS TX mapped by PyMOL. **(B)** The N-terminal Arg (R10), Asp (D12), Arg (R14), His (H36), and the mid-region Ser-Thr-Ser (S49-T50-S51) can bind with NAD^+^ to facilitate ADP ribosylation. **(C)** The C-terminal (Y571-F591) is integral to proper D3 folding for facilitating vacuolation.

The production and pathogenic activity of *M. pneumoniae* community-acquired respiratory distress syndrome toxin (CARDS TX) are mechanistically coupled with its adhesion process (Kannan et al., 2010). Substantial evidence reveals the coordinated upregulation of CARDS TX expression and key adhesins, with spatial colocalization observed at the terminal organelle. This functional synergy suggests that toxin deployment is spatially and temporally coordinated with host cell attachment, potentially amplifying cytotoxic effects through membrane-proximal action (Kannan et al., 2010; Techasaensiri et al., 2010). The expression of the CARDS TX gene, regulated by the mpn372 locus, serves dual functions as both a secreted cytotoxin and an adhesin. CARDS TX is a 591-amino-acid protein structurally divided into an N-terminal mART (D1 domain) and a C-terminal β-trefoil structure (D2 + D3 domain) (Figure 5A; Kannan et al., 2014; Becker et al., 2015).

CARDS TX facilitates host cell invasion through a dual-targeting mechanism that engages both protein receptors and membrane lipids. Notably, it can bind to surfactant protein-A (SP-A) and annexin A2 on the host cell surface, thereby initiating clathrin-mediated endocytosis (Kannan and Baseman, 2006). Furthermore, the C-terminal domains selectively interact with phosphatidylcholine (PC) and sphingomyelin (SM), lipid components enriched in membrane microdomains, thereby stabilizing bacterial adhesion and promoting toxin internalization (Becker et al., 2015). This coordinated engagement of receptors and lipids facilitates close bacterial-host interactions, enabling the localized delivery of CARDS TX at membrane interfaces and ultimately inducing cytotoxic effects (Kannan and Baseman, 2006; He et al., 2016).

As a virulence factor, *M. pneumoniae* exhibits both ADPRT and vacuolating activities. Key residues in the N-terminal (R10, D12, R14, H36) and mid-region (S49-T50-S51) bind NAD+ are critical for ADP ribosylation (Figure 5B; Krishnan et al., 2013; Kannan et al., 2014; Becker et al., 2015). As described above, this ADP ribosylation disrupts cell function (Kannan et al., 2014; Somarajan et al., 2014), leading to cytopathic effects (CPE) and eventual cell death (Figure 4; Krueger and Barbieri, 1995). The C-terminal region (Y571-F591) of CARDS TX mediates the binding of the toxin to the mammalian cell surface and induces subsequent endocytosis (Figure 5C; Krishnan et al., 2013; Kannan et al., 2014; Becker et al., 2015). Similar to the purified vacuolating cytotoxin (VacA) protein of H. pylori, internalization is essential for its vacuolation activity. CARDS TX-induced vacuoles are enriched with Rab9 GTPase, which mediates vacuole formation via the Golgi membrane (Johnson et al., 2011). While the vacuolation mechanism in *M. pneumoniae* remains unclear (Kannan and Baseman, 2006; Kannan et al., 2014; Somarajan et al., 2014; Becker et al., 2015), studies have highlighted similarities between CARDS TX and H. pylori VacA toxins (Table 2, Figure 4; Ansari and Yamaoka, 2020).

**TABLE 2:** The similarity between CARDS TX and *H. pylori* VacA toxins in terms of vacuolation.

Toxins	Protein structure	Receptors related to the internalization	Vacuolation
CARDS TX	Folded into three regions by 17α and 43β helices, it contains an N-terminal mART (D1 domain) and a C-terminal β-trefoil structure (D2 + D3 domain) in series.	SP-A, A2 protein, PC, SM	Rab9 enrichment occurred around the CARDS TX-induced vacuoles, and Rab9 was involved in vacuole formation through the Golgi membrane.
VacA toxins	An N-terminal p33 domain and a C-terminal p55 domain, linked by a flexible loop that is sensitive to limited proteolysis *in vitro*.	Tyrosine phosphatases (PRTPα and PRTPβ), lipoprotein receptor-related protein-1 (LRP1)	VacA is involved in the synthesis of large bacteria containing vacuoles in infected cells, caused by the fusion of late endocytic compartments.

### 3.2 The immune pathogenic mechanisms related to adhesion

#### 3.2.1 Inflammatory reaction related to adhesion

The adhesion ability of *M. pneumoniae* appears to be a prerequisite for activating the immune response during infection. Chaudhry et al. (2005) identified immunodominant regions in the C-terminus of P1 protein (residues 1,125–1,131 and 1,382–1,394) that trigger an immune response once the protein attaches to the mucosal surface. Hoek et al. (2005) demonstrated that adherent *M. pneumoniae* induces IL-4 production in murine basophils, whereas non-adherent strains fail to elicit significant cytokine responses. This disparity is primarily attributed to the higher abundance of P1 protein in adherent *M. pneumoniae*. Similarly, Shimizu et al. (2011) found that the wild-type strain of *M. pneumoniae* stimulates the production of inflammatory cytokines, including IL-1β and tumor necrosis factor-α (TNF-α). In contrast, a mutant strain lacking adhesion ability failed to induce cytokine production.

Besides, *M. pneumoniae* adheres to the host airway through LAMP, whose lipid portion is recognized by Toll-like receptor (TLR) complexes predominantly expressed on respiratory epithelial cells and immune cells (Malik et al., 2023), activating cellular signal transduction pathways (Zuo et al., 2009). Following adhesion, *M. pneumoniae* interacts with TLR4 to induce macrophage autophagy, enhancing the synthesis and secretion of pro-inflammatory cytokines, including IL-1β, IL-6, and IL-8 (Shimizu et al., 2014). Furthermore, the recognition of LAMP by TLRs triggers an inflammatory response characterized by lymphocyte, neutrophil, and occasionally eosinophil infiltration, promoting IL-1β, IL-6, IL-8, and TNF-α, as well as inflammatory mediators like ROS (Into et al., 2007; Shimizu et al., 2007, 2008; He et al., 2009).

#### 3.2.2 Immune escape related to adhesion

Although adhesion supports the survival of *M. pneumoniae* in the host, the immune response it triggers acts as a barrier to pathogenic progression. To counter this, *M. pneumoniae* adhesion proteins employ various immune evasion strategies to mitigate host defenses (Ferreira et al., 2006; Sluijter et al., 2009; Schmidl et al., 2010; Yu et al., 2020). After adhesion to epithelial cells, the adhesins of *M. pneumoniae* are phosphorylated by protein kinase PrkC, producing multiple phosphorylated forms that aid in immune evasion (Schmidl et al., 2010; Widjaja et al., 2020). Sluijter et al. (2009) demonstrated that the RecA protein homolog encoded by mpn490 promotes gene exchange between homologous DNA sequences (mainly RepMP) in *M. pneumoniae*, leading to variations in surface adhesins and facilitating immune evasion. Another study revealed that abundant complement factor H is expressed at the site of *M. pneumoniae* colonization, which strengthens adherence between mycoplasmas and tracheal epithelial cells. Moreover, *M. pneumoniae* can bind factor H through specific binding proteins, such as EF-Tu, PDH-B, and PDH-A, thereby mimicking host cells to regulate complement activation and evade immune responses (Ferreira et al., 2006; Yu et al., 2020).

## 4 Adhesion-related mechanisms in extrapulmonary infection

### 4.1 Direct adhesion-related pathogenic mechanism

**FIGURE 6:**
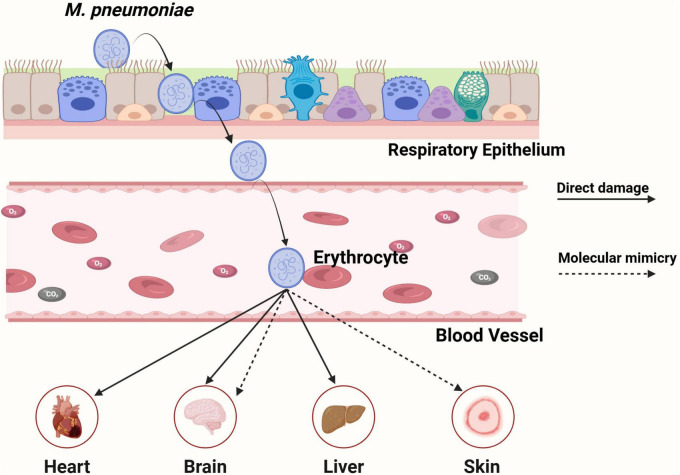
The adhesion-related mechanisms of *M. pneumoniae* in extrapulmonary infection.

*M. pneumoniae* infection also causes a wide range of extrapulmonary diseases involving multiple systems, including cutaneous, musculoskeletal, neurological, hematological, digestive, and renal systems (Hu et al., 2022). Direct adhesion has also been identified as a critical factor driving pathogen-host interactions, playing a significant role in the extrapulmonary pathogenesis. Beyond its well-established ability to bind respiratory epithelial cells, *in vitro* studies have also demonstrated that *M. pneumoniae* can adhere to macrophages and erythrocytes (Chaudhry et al., 2016; Poddighe, 2018). Notably, when colonizing respiratory surfaces with compromised epithelial integrity, particularly within immunologically immature or damaged mucosal barriers, the pathogen may exploit these adhesion sites and gain access to the systemic circulation through structural breaches in the epithelial layer (Choi et al., 2017). *M. pneumoniae* exhibits hemolytic activity through adhesion to erythrocyte membranes, mediated by interactions between bacterial surface proteins and SOS on red blood cells. These interactions alter erythrocyte surface antigens, promoting neo-antigen formation and molecular mimicry, which ultimately elicit complement-mediated autoimmune hemolysis (de Groot et al., 2017). *M. pneumoniae* adhesion to erythrocytes may further contribute to its extrapulmonary dissemination. Its successful isolation from pericardial effusion and cerebrospinal fluid provides clinical evidence supporting its capacity for direct tissue invasion (Neimark and Gesner, 2010). In addition, emerging clinical reports describe *M. pneumoniae*-associated hepatitis occurring before respiratory symptoms, suggesting a hepatotropic pathogenesis potentially mediated by direct bacterial adhesion to hepatic cells, although further evidence is required (Figure 6; Narita, 2016).

### 4.2 Indirect adhesion-related pathogenic mechanism

Current research remains limited in elucidating the extrapulmonary pathogenic mechanisms of *M. pneumoniae*, while molecular simulation approaches may offer new insights. Adhesion proteins localized at the *M. pneumoniae* terminal organelle, such as P1 and P30, exhibit C-terminal homology with host troponin and cytoskeletal proteins. This molecular mimicry enables cross-reactive antibodies against bacterial adhesins to bind host cytoskeletal components, thereby amplifying autoimmune responses (Dallo et al., 1990; Narita, 2010; He et al., 2016). Neurological manifestations represent one of the most prevalent extrapulmonary complications of *M. pneumoniae* infection. The pathogen’s P1 adhesin binds to membrane glycolipids to form galactocerebroside C (GalC)-like complexes, establishing structural homology with human myelin components. This molecular mimicry mechanism triggers the production of cross-reactive antibodies that mistakenly target myelin-associated glycolipids, such as GalC and gangliosides, leading to autoimmune-mediated demyelination. This mechanism underlies *M. pneumoniae*-associated encephalitis and Guillain-Barré syndrome (Meyer Sauteur et al., 2014a). Meanwhile, the molecular mimicry between the P1 adhesin of *M. pneumoniae* and keratinocyte antigens can induce the production of cross-reactive antibodies, formation of immune complexes, and complement activation, which collectively contribute to the development of Mycoplasma-induced rash and mucositis (MIRM) (Figure 6; Chaudhry et al., 2016).

## 5 The adhesion-related characteristics during *M. pneumoniae* asymptomatic carriage

Studies have shown that asymptomatic carriage of *M. pneumoniae* in the upper respiratory tract (URT) is common across all pediatric age groups. This high carriage rate complicates the diagnosis of *M. pneumoniae* infection, as the clinical symptoms and signs of respiratory tract infection are not specific and reliably predictive (Spuesens et al., 2013). Moreover, neither serological testing nor polymerase chain reaction (PCR) can reliably distinguish active infection from asymptomatic carriage, creating significant challenges for clinicians in determining whether and when to initiate antimicrobial therapy (Spuesens et al., 2014; Meyer Sauteur et al., 2016). In addition to diagnostic and therapeutic challenges, asymptomatic carriers of *M. pneumoniae* represent an important source of transmission. The carriage state has the potential to transition into symptomatic infection and can exacerbate pre-existing pulmonary conditions such as asthma, warranting heightened clinical attention (Spuesens et al., 2013; Koenen et al., 2023).

Considering the previously described adhesion pathogenesis, it is important to explore how the host maintains an asymptomatic carriage state for extended periods following initial colonization via bacterial adhesins. de Groot et al. (2022) reported that during asymptomatic carriage of *M. pneumoniae*, mucosal antibodies (IgG and IgA) targeting key adhesins of the bacterial attachment organelle, including P1, P30, and P116, are largely undetectable in the URT. This deficiency of specific mucosal antibodies may impair the host’s defense mechanisms against bacterial adhesion, as these immunoglobulins are typically involved in neutralizing adhesion-mediated interactions with epithelial surfaces. Consequently, persistent adherence facilitates long-term colonization and establishes an immunological niche that supports prolonged asymptomatic carriage. The failure to mount an effective mucosal antibody response may be attributed to the limited expansion and activation capacity of local B cells in the URT (Meyer Sauteur et al., 2018). In parallel, recent studies have demonstrated that asymptomatic carriage is associated with a marked reduction in URT microbiota diversity and an increased abundance of Haemophilus influenzae (Koenen et al., 2023). Such microbial dysbiosis, or the lack of normal microbial competition, may facilitate *M. pneumoniae* colonization. It is also well-established that natural immunity following *M. pneumoniae* infection is typically short-lived. Upon initial adhesion, *M. pneumoniae* forms a “firm but superficial” attachment to the epithelial surface. If the transient and relatively mild immune response fails to eliminate the pathogen, the host may transition into a prolonged asymptomatic carriage state once this short-lived immunity wanes (Waites et al., 2017). Notably, asymptomatic carriage may evolve into a self-perpetuating cycle. Persistent bacterial adhesion results in the continuous release of metabolic by-products and the induction of low-grade inflammation, which progressively damages the mucociliary barrier of the respiratory tract. This epithelial disruption impairs mucosal clearance, thereby promoting continued colonization. In turn, sustained colonization exacerbates epithelial damage and promotes chronic airway inflammation (Prince et al., 2018). Collectively, these factors contribute to a dynamic equilibrium between the pathogen and host, sustaining bacterial persistence without eliciting overt pathological responses (Meyer Sauteur et al., 2014b).

## 6 Advances in treatment and prevention based on the adhesion theory

### 6.1 Clinical significance of *M. pneumoniae* adhesion

Adhesion, as a key step in the pathogenic mechanism of *M. pneumoniae*, carries profound clinical significance. Without successful adhesion, the bacterium cannot initiate infection, as both direct cytotoxicity and immune activation are highly dependent on this process. To date, research on the clinical implications and patient outcomes related to *M. pneumoniae* adhesion has primarily focused on the P1 protein. Epidemiological studies have highly concentrated on P1 genotyping, which is based on sequence variation within the RepMP2/3 and RepMP4 repetitive elements. According to these variations, *M. pneumoniae* strains are broadly classified into P1-1 and P1-2 types (Spuesens et al., 2009; Lee et al., 2019). While the distribution of these P1 genotypes varies by geographic region and over time, current evidence suggests that P1 genotype alone does not reliably predict clinical outcomes, such as asymptomatic carriage, disease severity, or extrapulmonary manifestations (Meyer Sauteur et al., 2021). However, emerging data indicate that the P1-2 genotype is increasingly associated with high-level macrolide resistance and may exhibit a greater capacity for transmission compared to P1-1 strains (Li et al., 2022; Li et al., 2024). Therefore, continuous monitoring of P1 genotypes could offer valuable insights into antimicrobial susceptibility in pediatric populations and support more informed clinical decision-making. Additionally, advances in PCR-based denaturing gradient gel electrophoresis (DGGE) have significantly improved the sensitivity of detecting *M. pneumoniae* variants, which is potentially beneficial for patient prognosis and therapeutic outcomes (Xiao et al., 2014).

### 6.2 Current therapeutic strategies for *M. pneumoniae*

Although macrolides remain the first-line therapy for MPP, recent surveillance data indicate a rapidly increasing prevalence of MRMP strains worldwide (Jiang J. C. et al., 2021). *M. pneumoniae* synthesizes polypeptides by forming peptide bonds at the peptidyl transferase center (PTC) of the 50S ribosomal subunit and exporting them through the nascent peptide exit tunnel (NPET)—a critical step in bacterial protein synthesis. Previous studies have shown that macrolide antibiotics bind within the NPET, narrowing the tunnel’s diameter, thereby obstructing peptide elongation and inhibiting the synthesis of all nascent proteins, which underlies their antibacterial activity (Kannan et al., 2012; Lin et al., 2018). However, mutations at nucleotide positions A2063G and A2064G in the 23S rRNA, along with alterations in ribosomal proteins L4 and L22, hinder macrolide binding to the ribosome, leading to loss of drug efficacy (Pereyre et al., 2016). Furthermore, the recent predominance of the P1-2 genotype has been linked to the growing dissemination of MRMP strains. This rising threat of antimicrobial resistance highlights the urgent need for novel therapeutic strategies. Although tetracyclines (e.g., doxycycline) and fluoroquinolones (e.g., levofloxacin) are potential alternatives, their use in pediatrics is strictly restricted due to class-specific toxicity profiles. Tetracyclines are contraindicated in children under 8 years of age due to risks of permanent dental discoloration and enamel hypoplasia, while fluoroquinolones are associated with black-box warnings for musculoskeletal side effects, including tendinopathy and cartilage damage, primarily through matrix metalloproteinase inhibition (Spuesens et al., 2013). For patients with rapidly progressing disease or those experiencing severe complications, adjunctive therapies such as corticosteroids and gamma globulin have been employed. However, these agents primarily serve to suppress inflammation and do not exert direct antimicrobial effects (Gao and Sun, 2024). Given these limitations, there is increasing interest in the development of novel therapeutics based on the adhesion mechanisms of *M. pneumoniae*. Since adhesion is a key step in the bacterium’s pathogenesis, targeting this process may provide a promising alternative to conventional antimicrobial therapy and help overcome the challenges posed by macrolide resistance.

### 6.3 Treatment based on adhesion pathogenesis

In recent years, several compounds have been identified that effectively inhibit *M. pneumoniae* adhesion and show promising therapeutic potential. For example, Meng et al. reported that Platycodin D, a traditional Chinese medicine, significantly suppressed the expression of key adhesins P1 and P30. This led to bacterial detachment from respiratory epithelial cells, disrupted nutrient acquisition, and ultimately inhibited proliferation (Meng et al., 2017). Likewise, Galgowski et al. (2021) demonstrated that methanolic extract (ME) from Melipona quadrifasciata propolis, a stingless bee species endemic to Brazil, exhibits potent anti-adhesion activity against *M. pneumoniae*, underscoring the therapeutic promise of natural products in combating bacterial colonization. Besides, Reddy et al. (1996) evidenced that adhesins and adhesin-related accessory proteins of *M. pneumoniae* are proline-rich in composition and mediated successful adhesion to host target cells. Cyps facilitate bacterial adhesion by promoting the proper folding of proline-rich proteins involved in host cell attachment. However, their enzymatic activity can be selectively inhibited by cyclosporin A (CsA), thereby disrupting adhesion and offering a potential therapeutic strategy for MPP. Collectively, these findings highlight the potential of targeting *M. pneumoniae* adhesion pathways as an innovative approach to overcome the limitations of current therapies, particularly in light of increasing macrolide resistance.

### 6.4 Vaccine development based on adhesion pathogenesis

Since the discovery of *M. pneumoniae*, the development of effective vaccines has been a longstanding research focus; however, to date, no highly effective vaccine is available for the prevention of human *M. pneumoniae* infections (Zeng et al., 2025). Given the growing challenges posed by antimicrobial resistance and the limitations of current therapeutic strategies, there is an urgent need to develop a vaccine capable of protecting children from *M. pneumoniae* infections. Studies have shown that whole-cell inactivated vaccines exhibit limited efficacy, while live-attenuated vaccines raise significant safety concerns, both of which restrict their applicability in human populations (Jiang Z. et al., 2021). Consequently, attention has shifted toward subunit vaccine development, particularly focusing on adhesion-related proteins such as P1, P30, and P116, which have demonstrated strong immunogenicity and immunoreactivity (Jiang Z. et al., 2021). According to de Groot et al. (2022), during *M. pneumoniae* infection, the upper respiratory tract produces mucosal IgA and IgG antibodies that specifically target the adhesins P1, P30, and P116. These antibodies may provide a basis for vaccine strategies aimed at blocking bacterial adhesion, thereby disrupting infection and transmission and ultimately helping to reduce the burden of disease in children. In support of this approach, Schurwanz et al. (2009) identified the C-terminal region of P1 [amino acids (aa) 1288–1518] and the central region of P30 (aa 17–274) as highly immunoreactive and critical for host cell adhesion. They further designed a chimeric protein combining these regions, which induced monospecific antiserum that significantly reduced *M. pneumoniae* adherence to human bronchial epithelial cells (Schurwanz et al., 2009; Hausner et al., 2013). Additional evidence suggests the potential of mRNA vaccine technology in this context. Zeng et al. demonstrated that anchoring the C-terminal domain of the P1 adhesin to an mRNA vaccine conferred partial protection against *M. pneumoniae* infection in animal models (Zeng et al., 2025). Similarly, Svenstrup et al. purified the P116 protein and observed that polyclonal antibodies targeting P116 effectively inhibited bacterial adhesion to HEp-2 cells, highlighting its value as a vaccine candidate (Jiang Z. et al., 2021). Therefore, all the antigens above have the potential to become antigenic candidates for vaccine research. In 2016, Chen et al. (2016) designed a chimeric protein (P116N-P1C-P30), named MP559, which contains multiple antigenic epitopes of the above three antigens. Vaccination with MP559 stimulated the same humoral immune response as vaccination with these three antigens alone. This study showed that MP559 has the potential to replace the three individual subunit vaccine candidate proteins. Despite the encouraging immunogenicity observed in animal models, none of these vaccine candidates have advanced to clinical trials. Their efficacy and safety in humans remain significant hurdles. Moreover, vaccine-enhanced disease (VED) has emerged as a critical concern in *M. pneumoniae* vaccine development. LAMP from *M. pneumoniae* has been implicated in exacerbating inflammatory responses. In animal studies, mice vaccinated with LAMP exhibited more severe inflammation and tissue pathology compared to controls, suggesting that careful consideration of LAMP components is necessary during vaccine formulation (Mara et al., 2020; Mara et al., 2022).

## 7 Conclusion

Adhesion-related pathogenesis plays a central role in *M. pneumoniae* infection. By attaching to respiratory epithelial cells through its specialized terminal organelle and various adhesion proteins, *M. pneumoniae* initiates its infectious process. The gliding motility further enhances bacterial colonization and spread. Once adhered, *M. pneumoniae* exploits host-derived nutrients and releases cytotoxic substances such as H_2_O_2_ and CARDS TX, leading to host cell damage. In parallel, bacterial adhesion triggers robust inflammatory responses and facilitates immune evasion by masking surface antigens, thereby prolonging bacterial survival within the host. Notably, adhesion-mediated mechanisms are also implicated in extrapulmonary manifestations, contributing to the broad clinical spectrum of *M. pneumoniae* infection. Due to the core pathogenic mechanism of adhesion, some treatments and preventions of *M. pneumoniae* infections targeting adhesion are currently some of the research hotspots.

Despite these insights, key questions remain unanswered, particularly regarding the differences in adhesion dynamics between symptomatic infections and asymptomatic carriage. Understanding these distinctions is critical for developing targeted interventions.

Looking ahead, future research should aim to further clarify the molecular mechanisms of *M. pneumoniae* adhesion and gliding, identify therapeutic targets within host-pathogen interactions, and assess immune responses to adhesion disruption. Clinically, combining adhesion-targeted strategies with existing antibiotics may enhance treatment efficacy, reduce transmission, and help curb resistance. Adhesion-based vaccines targeting immunogenic regions of these proteins offer a promising approach to prevention, especially amid rising macrolide resistance. Continued research into adhesion mechanisms will be essential for developing more effective therapies and preventive measures across all age groups.
